# On the Unique Morphology and Elastic Properties of Multi-Jet Electrospun Cashew Gum-Based Fiber Mats

**DOI:** 10.3390/polym16101355

**Published:** 2024-05-10

**Authors:** Mattia Grumi, Cristina Prieto, Roselayne F. Furtado, Huai N. Cheng, Atanu Biswas, Sara Limbo, Luis Cabedo, Jose M. Lagaron

**Affiliations:** 1 Novel Materials and Nanotechnology Group, Institute of Agrochemistry and Food Technology (IATA), Spanish Council for Scientific Research (CSIC), Calle Catedrático Agustín Escardino Benlloch 7, 46980 Paterna, Spain; mgrumi@iata.csic.es; 2 Embrapa Agroindústria Tropical, Rua Dra. Sara Mesquita 2270, Fortaleza 60511-110, Brazil; roselayne.furtado@embrapa.br; 3 U.S. Department of Agriculture, Agriculture Research Service, Southern Regional Research Center, 1100 Allen Toussaint Blvd., New Orleans, LA 70124, USA; hncheng100@gmail.com; 4 U.S. Department of Agriculture, Agricultural Research Service, National Center for Agricultural Utilization Research, 1815 N. University St., Peoria, IL 61604, USA; abiswas1955@yahoo.com; 5 Department of Food, Environmental and Nutritional Sciences (DeFENS), Università degli Studi di Milano, Via Giovanni Celoria 2, 20133 Milan, Italy; sara.limbo@unimi.it; 6 Polymers and Advanced Materials Group (PIMA), Universitat Jaume I (UJI), 12006 Castellon, Spain; lcabedo@uji.es

**Keywords:** cashew gum, nanofibers, electrospinning

## Abstract

This study investigates the unique morphology and mechanical properties of multi-jet electrospun cashew gum (CG) when combined with high-molecular-weight polyethylene oxide (PEO) and glycerol. Cashew gum (CG) is a low-cost, non-toxic heteropolysaccharide derived from *Anacardium occidentale* trees. Initially, the electrospinnability of aqueous solutions of cashew gum alone or in combination with PEO was evaluated. It was found that cashew gum alone was not suitable for electrospinning; thus, adding a small quantity of PEO was needed to create the necessary molecular entanglements for fiber formation. By using a single emitter with a CG:PEO ratio of 85:15, straight and smooth fibers with some defects were obtained. However, additional purification of the cashew gum solution was needed to produce more stable and defect-free straight and smooth fibers. Additionally, the inclusion of glycerol as a plasticizer was required to overcome material fragility. Interestingly, when the optimized formulation was electrospun using multiple simultaneous emitters, thicker aligned fiber bundles were achieved. Furthermore, the resulting oriented fiber mats exhibited unexpectedly high elongation at break under ambient conditions. These findings underscore the potential of this bio-polysaccharide-based formulation for non-direct water contact applications that demand elastic properties.

## 1. Introduction

Plastic pollution and a lack of biodegradability, together with the scarcity of oil sources, have boosted worldwide research into renewable and biodegradable alternatives to petroleum-based polymers for various applications [1]. In this connection, agro-based polymers and natural gums have been described as promising biodegradable polymeric materials [2]. The term “gum” identifies exudates or slims, which are not part of the vegetable cell wall, also known as polysaccharide hydrocolloids [3]. The biocompatibility, low risk of adverse effects, low cost, and their environmentally friendly processing are among the advantages attributed to natural gums [4,5]. Natural gums have been traditionally used as clarifying and gelling agents, emulsifiers, thickeners, and stabilizers in the food industry [6].

Cashew gum, a complex water-soluble heteropolysaccharide, is extracted from the bark epithelial cells of *Anacardium occidentale* Linn trees, commonly known as the cashew trees [7]. These trees are mainly located in tropical and subtropical countries, even though their cultivation is, up to now, mostly aimed at the production of cashew nuts, while cashew gum was traditionally considered a low-value by-product [8]. Cashew gum can be obtained by spontaneous exudation, since it is synthesized in the epithelial cells and secreted, as a response to mechanical stimuli or attacks by pathogens, into ducts through the *Anacardium occidentale* tree bark [5,9], or it can be collected by means of incisions on the trunk and branches of the tree. The extraction can also be promoted by the use of chemical stimulants [10]. Cashew gum structure is characterized by a highly branched galactan arrangement composed of branched chain of ß-(1–3)-linked D-galactose residues interspaced with ß-(1–6) linkages [11,12], in which the main residues are represented by galactose (59–73%), glucose (6–14%), arabinose (4–5%), rhamnose (2–4%) and glucuronic acid (6–14%) [13,14]. Among the potential cashew gum applications, several studies have been conducted revealing its huge potential in different sectors such as food, pharma, and food packaging, among others [15,16]. Moreover, it can be asserted that cashew gum is a non-toxic, biocompatible, inexpensive, and biodegradable natural polymer with good mechanical and rheological properties [14,15], easily soluble in water, and with interesting adhesive, stabilizing, and emulsifying properties [10,17]. Cashew gum also has functional properties such as mucoadhesion, anti-inflammatory, anti-diarrheal, anti-microbial, anti-tumoral and hypoglycemic effects [16]. All these properties suggest that cashew gum is a suitable candidate for multiple purposes such as food, pharma, biomedicine, cosmetics, agriculture, or food packaging applications.

Among the available processing technologies discovered during the last two decades, a growing interest has been focused on the novel electrohydrodynamic processing technology (EHDP) that stands out as a simple and versatile approach for the production of ultrathin (micron and submicron) polymer structures with highly specific surface areas and porosities for several industrial applications, including the manufacture of food packaging materials [18,19]. In the electrospinning process, a polymeric solution is exposed to a high electric field, which allows its surface tension to be overcome, enabling an electrically charged fluid stream of the polymeric phase to be ejected towards the collector [20]. During the “flight time”, the solvent evaporates at room temperature and the jet undergoes continuous elongation, allowing the creation of widely interconnected micro- or nanofibers that generate a three-dimensional network in the non-woven electrospun mat [21]. The obtained ultrathin structures provide several functional benefits, such as tailor-made porosity, enhanced surface-to-volume ratio, adaptable morphology, unique mechanical properties [22,23], and the ability of encapsulation of active and bioactive compounds and their subsequent release [24,25].

The contribution of this study was the development and characterization of electrospun fibers of cashew gum, which required assistance by PEO and glycerol to exhibit unique morphology and mechanical properties. The interest and selection of these materials is that they pose no toxicity and are used in food, medical, and pharmaceutical applications. Cashew gum was previously studied by Vázquez-González et al. [16], who assessed the electrosprayability of cashew gum to form capsules. Vazquez-Gonzalez et al. [26], in a recent study also carried out in our lab, reported the use of one of the mixtures of a similar cashew gum and high-molecular-weight PEO and also of other biopolymers, whose development process is presented in this paper, to encapsulate *M. caribbica* to fight fungal activity in avocado. Another recent study also assessed an electrospun blend of cashew gum and polyvinyl alcohol (CG-PVA) nanofibers, with an optimal ratio of 7:3, for scarless wound healing [27]. Andrade et al. [10] studied the thermoplastic extrusion of cashew gum to determine the effect of this processing technique on this natural gum. Furthermore, Oliveira et al. [28] and Silva et al. [9] studied the formation of cashew gum-based films by casting or by blending the biopolymer with gelatin or nanofibrillated bacterial cellulose. Recently, de Souza et al. [29] successfully blended cashew gum with alginate, galactomannans, and gelatin in order to produce edible coatings for food preservation.

In this study, both low- and high-throughput electrospinning of water-based solutions with high content of cashew gum combined with minor contents of high-molecular-weight PEO and glycerol were carried out, and the resulting morphological and mechanical properties were characterized for the first time. The developed formulation showed an unexpected and unreported elasticity during tensile tests when subjected to multi-jet electrospinning, potentially representing a sustainable alternative to conventional non-biobased elastomers.

## 2. Materials and Methods

### 2.1. Materials

Non-purified cashew gum (*Mw*: 2.13 × 10^4^ Da) was obtained from native *Anacardium occidentale* L. trees cultivated by Embrapa Tropical Agroindustry (Fortaleza, CE, Brazil), and polysaccharide isolation from cashew gum (CG) was carried out following the procedure previously determined by da Silva et al. [30]. Polyethylene oxide (PEO) (*Mw*: between 6.0 × 10^5^ Da to 5.0 × 10^6^ Da) and Span^®^ 20 surfactant were acquired from Sigma Aldrich (St. Louis, MO, USA). Pure glycerol (*Mw*: 9.21 × 10^1^ Da) was provided by Panreac AppliChem (Barcelona, Spain). Distilled water was used as the only solvent throughout this work. All chemicals were used with no further purification.

### 2.2. Preparation of Polymeric Solutions

For the evaluation of the electrospinnability of CG, different formulations of CG in distilled water were prepared as shown in Table 1. CG, at a concentration of 120% (*w*/*v*), was first dissolved under magnetic stirring in distilled water for 48 h at room temperature (i.e., 25 °C), and Span^®^ 20 was added at a concentration of 3% (*w*/*v*). This corresponds to solution *S0*.

For the purpose of improved fiber formation, blending with a higher-molecular-weight supporting polymer was considered. In this case, blending with PEO of different molecular weights was proposed. Total solid concentration in solution (TCS) varied from 50% to 15% (*w*/*v*). The CG:PEO ratio varied from 95:5 to 82:18. For the preparation of the solutions, CG was first dissolved in distilled water and left under magnetic stirring for 48 h at room temperature (i.e., 25 °C). Then, PEO was aggregated into the cashew gum solution and further stirred for at least 3 h. In all polymeric solutions (*S1*–*S8*), Span^®^ 20 was added at 1% (*w*/*v*) with respect to the volume of distilled water used.

In further tests, the removal of some impurities of cashew gum was undertaken to improve the stability of the electrospinning process. Thus, two consecutive centrifuging steps were conducted after the complete solubilization of CG in distilled water. Each centrifuging step was performed for 20 min at 11,872× *g* using a J-26 XPI Beckman Avanti equipment with a JA-25.50 rotor (Beckman Coulter Life Sciences, Indianapolis, IN, USA). The supernatant without impurities was recovered and PEO was added to the solution. Then, Span^®^ 20 was added to the solution at 1% (*w*/*v*) with respect to the volume of distilled water. The obtained polymeric solution was gently homogenized under magnetic stirring at 35 °C for 4 h (solution *S9*). In this solution, a final CG:PEO ratio of 82:18 was achieved.

For three formulations, a plasticizer, in this case glycerol, was used at concentrations of 1.5–3.5% (*w*/*v*) and added to the CG solution after the complete solubilization of PEO, and it was further stirred for at least 3 h in order to guarantee homogeneity (solutions *S10*–*S12*).

Three different polymeric solutions were additionally prepared and used as controls to better comprehend the specific morphology and properties of the multi-jet electrospun *S12* optimal blend. Details of the composition of these solutions, *Control_1* to *Control_3*, are gathered in Table 1.

### 2.3. Physicochemical Characterization of the Solutions

The viscosity, electrical conductivity, and surface tension of the polymeric solutions were measured before the electrospinning process. The solution viscosity was calculated using a ROTAVISC lo-vi control rotational viscosimeter (IKA-Werke GmbH & Co. KG; Staufen, Germany) equipped with an “sp-2” spindle rotating at 1.0 rpm. The electrical conductivity was assessed with an HI-4521 multi-parameter potentiometer (Hanna Instruments, Melrose, MA, USA). The surface tension was analyzed in an EasyDyne-K20 tensiometer (Krüss GmbH, Hamburg, Germany) following the Wilhelmy Plate procedure. All measurements were executed in triplicate and at room conditions.

### 2.4. Electrospinning Process

Polymeric solutions were electrospun in Fluidnatek^®^ LE-50 equipment (Bioinicia S.L., Valencia, Spain). Each prepared solution was drawn in a plastic syringe that was then inserted into an infusing syringe pump and connected by means of a polytetrafluoroethylene (PTFE) tube to a single injector linked to a stainless steel needle. The injector was coupled with a positive electrode at a high voltage. Different operational parameters, viz., voltage, distance between injector and collector, flowrate, and needle gauge, were tested in order to optimize fiber production. The applied flowrate ranged from 50 to 450 µL/h. A tip-to-collector distance, from 17 to 30 cm, was used. The applied positive voltage ranged from +17 to +31 kV. In addition, the applied negative voltage was always set at −9 kV when it was required. The needle gauge used was in the range between 22 and 27. All the trials performed to attain optimal fibers were conducted at 25 °C and 40% relative humidity (RH).

Besides the above single-needle experiments, the selected *S12* sample and *Control_1*–*Control_3* were produced in a higher-throughput mode using a Fluidnatek^®^ LE-100 electrospinning machine (Bioinicia S.L., Valencia, Spain) to obtain a wider homogeneous sample area to test for mechanical properties. In this case, the positive and negative voltage were optimally set at +15 and −20 kV, respectively; the tip-to-collector distance was set at 24.5 cm; the flowrate was set at 1 mL/h per needle, using a 5-needle injector (the injector setup consisted of 5 needles placed in a row with a distance between the needles of 13 mm) over a drum collector with a rotational speed of 100 rpm (collector diameter: 110 mm; linear speed: 0.58 m·s^−1^); the X axis scanning motion was set to cover the whole width of the drum collector (300 mm) using a motion speed of 50 mm/s. The needle gauge used was 23. This experiment was optimally conducted at 40 °C and 25% RH to achieve a better solvent evaporation while electrospinning the water-based polymeric solution *S12*. All the samples were stored for at least 24 h in a desiccator at 25 °C and a controlled 0% RH to preserve their integrity until further analysis.

### 2.5. Characterization of the Electrospun Fibers

#### 2.5.1. Scanning Electron Microscopy (SEM)

The morphology of the electrospun fibers was analyzed by scanning electron microscopy (SEM) using a Hitachi S-4800 microscope (Hitachi High-Technologies Corporation, Tokyo, Japan). Each sample, sized approximately 5 mm × 5 mm, was placed on the SEM sample holder using a double-side tape. All the samples were coated with a gold/palladium alloy for 2 min using a Polaron sputter coater (Quarum Technologies, Kent, UK) before the analysis. During the SEM analyses, a 10 kV electron beam acceleration was applied. Average fiber diameter was determined using the software ImageJ bundled with Zulu OpenJDK Version 13.0.6 (National Institute of Health, Bethesda, MD, USA), from the measurement of a minimum of 50 fibers.

#### 2.5.2. Thermogravimetric Analysis (TGA)

For the assessment of the thermal stability of PEO, cashew gum, and the electrospun cashew gum/PEO-based fibers obtained from *S12*, TGA was executed under nitrogen atmosphere in a 550-TA thermogravimetric analyzer (TA Instruments, New Castle, DE, USA). All TGA tests were performed in triplicate. TGA curves were acquired after conditioning the samples placed in the sensor at 30 °C for 5 min. After that, the samples were heated from 30 °C to 600 °C at a rate of 10 °C/min. Derivative TGA curves (DTGs), obtained using the TA analysis software TRIOS, Version 5.1 (TA Instruments, New Castle, DE, USA), expressed the weight loss rate as a function of temperature.

#### 2.5.3. Differential Scanning Calorimetry (DSC)

Thermal transitions of PEO and CG powder as well as those of the electrospun CG/PEO-based fibers obtained from *S12* were analyzed using a DSC-8000 analyzer (PerkinElmer Inc., Waltham, MA, USA) coupled with an Intracooler 2 cooling accessory (PerkinElmer Inc., Waltham, MA, USA). Approximately 3 mg of sample was inserted and hermetically sealed into an aluminum capsule, while an empty capsule was used as a reference. A calibration step was previously executed using an indium sample. The samples were first heated from 30 °C to 175 °C, then cooled back to 0 °C, and then heated back up again to 245 °C. The heating and cooling rates were set at 10 °C/min. The analyses were conducted under a nitrogen atmosphere and all DSC tests were performed in triplicate. The melting temperature (*T*_m_) and enthalpy of melting (Δ*H*_m_) values were derived from the heating scans, while the crystallization temperature from the melt (*T*_c_) and enthalpy of crystallization (Δ*H*_c_) were extracted from the cooling scans.

#### 2.5.4. Wide-Angle X-ray Scattering (WAXS)

The crystallographic structural analysis of PEO, CG, and the electrospun CG/PEO-based fibers obtained from *S12* was performed by WAXS in a WAXS D4-Model Endeavor diffractometer (Bruker Corporation, Ettlingen, Germany), following the same protocol previously reported by Vázquez-González et al. [31]. The difractograms were taken at room conditions and at scattering angles (2θ) between 5 and 30°. Reflection mode was used and incident Cu K-alpha radiation (Cu Kα = 1.54 Å) was applied. The generator was running at an operating voltage of 40 kV and a 40 mA filament current.

#### 2.5.5. Mechanical Tests

Tensile tests of electrospun fibers from *S12*, *Control_2* fibers, and *Control_3* fibers were performed following the ASTM standard method D638 using an AGS-X 5000 N universal testing machine (Shimadzu Corporation, Kyoto, Japan) provided with a 500 N load cell. Dumb-bell-shaped samples were die-cut from the fiber mat in both the direction of rotation of the drum collector (RD) and in the perpendicular direction (TD). The tests were performed under room conditions, i.e., 25 °C and 45% RH, at a 10 mm/min crosshead rate. Prior to the tensile assay, samples were conditioned to the test conditions for 24 h. A minimum of six die-cut specimens were measured for each sample.

#### 2.5.6. Statistical Analysis

Each treatment was carried out in triplicate and an evaluation of the results with a 95% significance level (*p* ≤ 0.05) was conducted. In order to identify significant differences among the treatments, Analysis of Variance (ANOVA) and Tukey multiple comparison test were realized. To this end, the software Origin Pro, Version 2023 (OriginLab Corporation, Northampton, MA, USA) was utilized.

## 3. Results and Discussion

### 3.1. Fiber Production and Physicochemical Characterization of the Solutions

The ability of CG to form fibers via electrospinning was first evaluated alone and also in combination with different mass ratios of higher-molecular-weight supporting polymers such as polyethylene oxide (PEO). In the latter case, the objective was to obtain cashew gum fibers with the smallest amount of PEO possible. The viscosity, electrical conductivity, and surface tension of the polymeric solutions were assessed before the electrospinning process in order to anticipate the stability of the process itself. Table 2 displays the characterization of the solutions with the best electrospinning performance. Solution *S8*, which was made using CG and PEO_5M_ solubilized in distilled water, showed a viscosity of 24,647 ± 148 cP, whereas solution *S9* presented a viscosity value of 21,849 ± 197 cP, which was significantly lower than the viscosity value obtained for solution *S8*. This decrease in the viscosity value could be due to the removal of insolubilized GC particles during the centrifuging process, which were present in solution *S8*. Similar observations in terms of viscosity reduction were reported previously [32] when purifying polymeric solutions based on unpurified PHBV by centrifugation. However, an increase in solution viscosity was observed for solution *S12*, which showed a viscosity of 24,189 ± 115 cP, as a consequence of the addition of 3.5% *w*/*w* of glycerol. This effect was previously described by Akinalan Balik et al. when studying the electrospinnability of pectin due to the addition of glycerol [33]. The latter authors observed an increase in viscosity even using a lower quantity of glycerol, i.e., 3% wt.

As regards conductivity, solution *S8* presented the highest conductivity. In fact, the reduction in the conductivity value observed in solution *S9* and *S12* may be ascribed to the removal of some salts and ions by centrifuging the solubilized cashew gum, which facilitates a more stable electrospinning process since solutions with high electrical conductivity values often suffer from process instability [34].

In terms of surface tension, it is important to indicate that the addition of Span^®^ 20 was required for all the solutions in order to decrease the surface tension value and obtain a stable electrospinning process. Since the solvent used was distilled water, which features a high surface tension value (around 70 mN/m) [35], the addition of a surfactant, such as Span^®^ 20, allowed a surface tension reduction down to values required for a good electrospinning process (around 40 mN/m) [36,37]. In view of the fact that the surfactant concentration was about the same (1% *w*/*v*) in these solutions, no significant differences were observed between these three samples.

### 3.2. Morphology of Electrospun CG-PEO Fibers

The electrospinning of the neat CG solution (solution *S0*) did not result in the production of consistent fibers. Thick structures were collected instead, as can be seen from Figure S1 included in the Supplementary Materials.

All the prepared CG-PEO solutions were intended to find the minimum amount of supporting polymer necessary to generate the required molecular entanglements to produce the desired electrospun fibers. However, solution *S5* was found not to be electrospinnable due to its excessively high viscosity [38], and hence it could not be processed and was excluded from the study. On the other hand, it was possible to electrospin solutions *S1*, *S2*, *S3*, *S4*, *S6*, *S7*, *S8*, *S9*, *S10*, *S11*, and *S12*, and their optimal processing conditions are summarized in Table 3.

**Figure 1:**
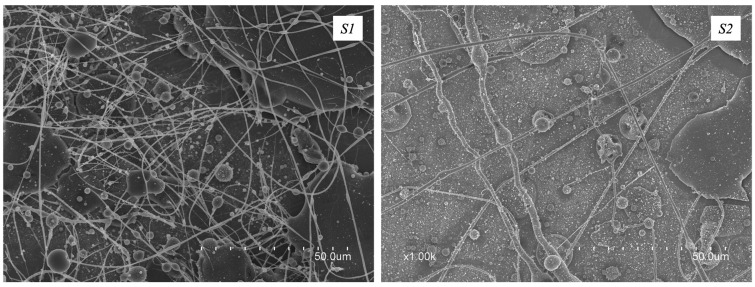
SEM micrographs of the electrospun fibers obtained from solutions *S1* and *S2*.

Figure 1 displays the micrographs of the electrospun fibers from solutions *S1* and *S2*, having a total concentration of solids of 50% *w*/*v*, and being produced with PEO molecular weights of 6 × 10^5^ and 1 × 10^6^ Da. The solution *S1*, as can be appreciated from Figure 1, led to the production of beaded fibers. A similar result was obtained with solution *S2*. For this reason, we decided to use a higher-molecular-weight PEO and to decrease the total concentration of solids up to a value of 20% *w*/*v*.

**Figure 2:**
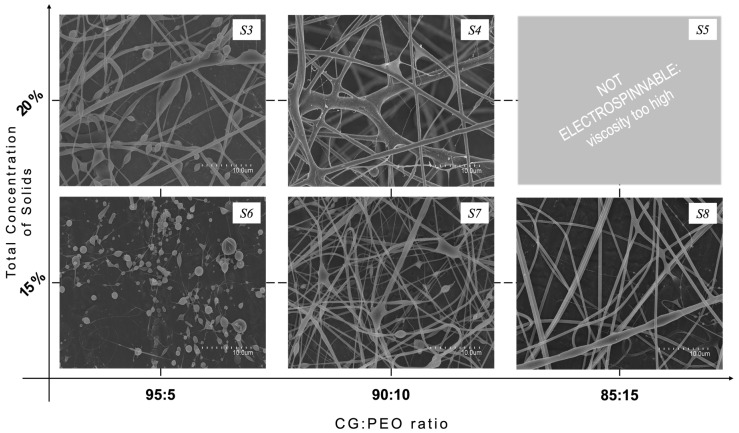
SEM micrographs of the electrospun fibers obtained from solutions *S3*–*S8*.

In solutions *S3*, *S4*, and *S5*, PEO with a molecular weight of 5 × 10^6^ Da (PEO_5M_) was used and the total solid concentration was decreased to 20% *w*/*v*. The experimented CG:PEO ratios were 95:5, 90:10, and 85:15, respectively. Solution *S3* allowed the production of fibers with a better morphology compared to those obtained from solution *S1*, as can be appreciated from observation of Figure 2, but still presented a considerable number of beads. The bead formation could be related to the viscoelastic nature of the polymeric solution, as well as to the processing conditions [39]. By increasing the ratio of PEO (solution *S4*), more homogeneous fibers were obtained compared to those obtained with solution *S3*, even if some wet fibers were seen, probably due to processing instabilities from the presence of some impurities. As previously reported, it was not possible to electrospin solution *S5*, probably due to its high content of PEO_5M_ that generated excessive viscosity.

Additionally, a lower total solid concentration of 15% *w*/*v* was explored, with CG:PEO_5M_ ratios of 95:5, 90:10, and 85:15, respectively, being identified as solutions *S6*, *S7* and *S8*. Solution *S6* did not result in the production of fibers, as can be observed from Figure 2, and only particles were produced. Increasing the PEO_5M_ content allowed the production of fibers but still showed some spindle-like defects, and wet fibers were also collected. The CG:PEO_5M_ ratio of 85:15 (Solution *S8*), on the contrary, allowed the formation of smooth fibers that were almost defect-free, as shown in Figure 2. The average diameter of these fibers was 0.9 ± 0.2 μm.

**Figure 3:**
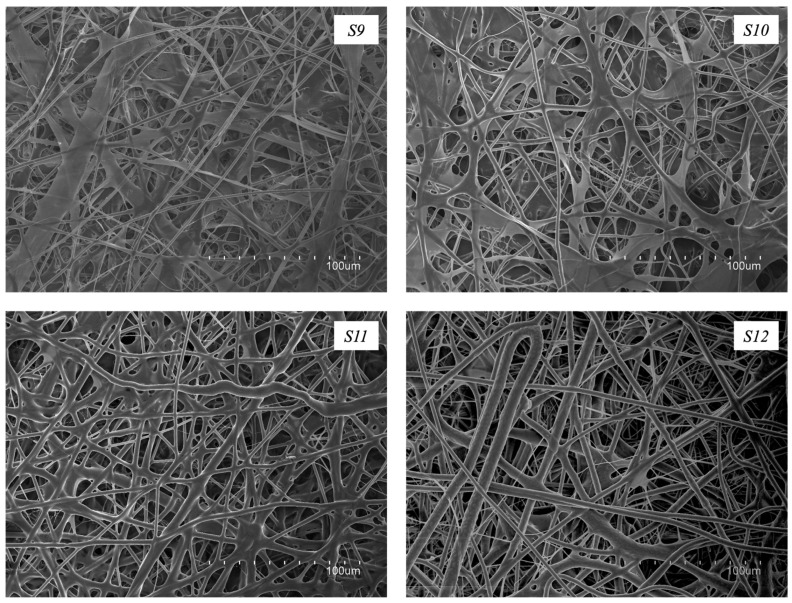
SEM micrographs of the electrospun fibers obtained from solutions *S9*–*S12*.

In an effort to improve morphology further, a centrifuging step after CG solubilization was performed to remove some CG impurities (solution *S9*). As shown in Figure 3, this strategy allowed the production of fiber mats with an enhanced morphology and completely free of defects. However, the fiber mats obtained were deemed too fragile to be handled. For this reason, the incorporation of a plasticizer, such as glycerol, was considered. Furthermore, Figure 3 shows how the different amounts of glycerol added to the centrifuged CG:PEO_5M_ solution affected the electrospun fibers’ morphology. Solutions *S10* to *S12* were related to fibers containing 1.5%, 2.5%, and 3.5% *w*/*w* of glycerol, respectively. The use of the plasticizer led to the production of defect-free electrospun fibers with enhanced morphology. Similar observations were reported previously [33] when studying the electrospinnability of pectin, also using glycerol as a plasticizer. Solution *S12* was chosen as the optimal formulation, namely the solution that led to producing the best fibers in terms of morphology, but also in terms of reduced fragility, especially when compared to the fibers obtained from solutions *S8* and *S9*, and any further post-treatment would not be needed. Further studies will continue to be focused on this formulation. Fibers obtained with solution *S12* presented an average fiber diameter of 3.1 ± 1.0 μm. The increase in fiber diameter observed in comparison with fibers obtained from solution *S8* could be due to the increase in viscosity produced as a consequence of the centrifugation process and the addition of glycerol, as reported before. Similar results were observed by Melendez-Rodriguez et al. [32], who observed a significant increase in the diameter of the unpurified PHBV-based fibers after a centrifuging step, suggesting a clear improvement of the polymer purity, together with an increase in the viscoelastic properties of the electrospun material, which led to the production of fibers with increased diameters [40]. In another study conducted by Akinalan et al. [33], the addition of glycerol led to the production of significantly thicker pectin-based electrospun fibers, probably due to the plasticizing action of glycerol which facilitated the molecular entanglement of the polymer chains. An analogous phenomenon was also reported previously [41], where the utilization of glycerol resulted in electrospun fibers with larger diameters compared to those where the glycerol was not used.

### 3.3. Thermal Properties and Thermal Stability

#### 3.3.1. Differential Scanning Calorimetry (DSC)

**Figure 4:**
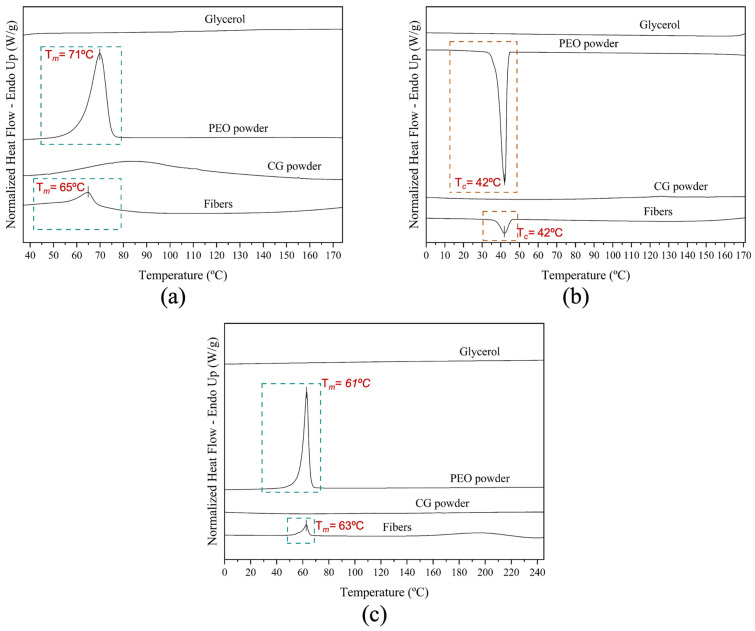
Typical differential scanning calorimetry (DSC) curves of the electrospun fibers from solution *S12* (Fibers), the pure cashew gum (CG), pure PEO_5M_ (PEO), and the pure glycerol. DSC curves are reported during (**a**) first heating step, (**b**) cooling step, and (**c**) second heating step.

Thermal transitions of electrospun fibers from solution *S12*, neat CG powder, neat PEO_5M_ powder, and pure glycerol were investigated through differential scanning calorimetry (DSC). The DSC curves are shown in Figure 4.

The DSC curve of neat CG powder did not show any melting or crystallization events in the distinct heating and cooling steps, which may be related to the amorphous structure of the biopolymer [33,42].

The DSC curve of the neat PEO powder during the first thermal step exhibited an endothermic peak at 71 °C (*T*_m_) with an Δ*H*_m_ of 202 J/g (Figure 4a), corresponding to its melting event. Comparable results were also reported previously [43] when studying the crystallization kinetics of PEO and its blend with poly(bisphenol A-co-epichlorohydrin). During the second heating run, which was carried out up to higher temperatures (250 °C), a similar endothermic peak, but at 61 °C (*T*_m_), with an Δ*H*_m_ of 176 J/g, was reported. Similar results were also reported by Medeiros et al. [44]. During the intermediate cooling step between the thermal runs, the DSC curve of neat PEO powder showed an exothermic peak at 42 °C (*T*_c_) with Δ*H*_c_ of −170 J/g. Kanis et al. reported a crystallization temperature at 53 °C [45]. The DSC curve of neat glycerol did not show any enthalpic features.

The CG:PEO fibers produced with solution *S12* showed clear melting and crystallization peaks associated with the PEO phase. The enthalpies were normalized by the PEO content in the blend, since it was the only crystallizable polymer [46]. In the first heating step, the fibers showed one endothermic peak at 65 °C (*T*_m_), with an Δ*H*_m_ of 158 J/g, ascribed to the PEO phase [31]. A reduction in both melting temperature and enthalpy was observed when compared with pure PEO. This could be attributed to an impairment in crystallinity development due to the so-called “dilution effect” [47] induced by the presence of CG, but also due to the presence of glycerol [48].

The crystallization temperature (*T*_c_) and enthalpy of the PEO phase were found at 42 °C and −131 J/g, respectively. During the second heating run, the fibers showed an endothermic peak at 63 °C ™, with an Δ*H*_m_ of 124 J/g. In this second heating step, the peak ascribed to the PEO phase presented a broader endothermic melting peak with a lower temperature shoulder. The observed reduction in enthalpy compared to the neat PEO was also reported by Cohen and Rocco [43], and they related it to a slower crystallization in the blend, indicating that it was more difficult for PEO crystals to nucleate and grow. A similar behavior of comparative PEO phase enthalpy reduction was also described by Vázquez-González et al. [31] when studying the thermal properties of fibers based on the FucoPol polysaccharide-PEO blend produced by electrospinning.

#### 3.3.2. Wide-Angle X-ray Scattering (WAXS)

**Figure 5:**
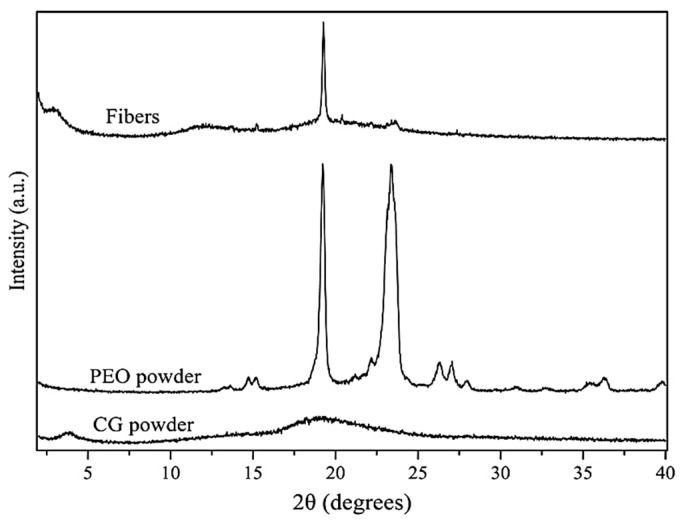
Wide-Angle X-ray Scattering (WAXS) patterns of neat cashew gum, neat PEO_5M_ (PEO), and fibers obtained from solution *S12* (fibers).

The crystalline phase of materials is often studied with the Wide-Angle X-ray Scattering (WAXS) technique. WAXS was used to characterize the crystalline structure of the neat CG, neat PEO_5M_, and the fibers obtained from solution *S12*. The WAXS diffractograms are presented in Figure 5.

The diffractogram of PEO_5M_ showed multiple peaks due to its semicrystalline nature, with two intense peaks at 24.0 and 19.8°, which were particularly distinctive. Similar results, in the 2θ range of PEO_5M_, were also found in other studies where two characteristic diffraction peaks at 23.5° and 19° were clearly visible [49] and were attributed to the (112) and (120) crystallographic planes of PEO_5M_, respectively [50]. Weak crystalline peaks at 2θ values around 15°, 26°, and 36° were also described by Lin et al. and Sunderrajan et al. [51,52], confirming the results obtained in this study.

On the contrary, it was not possible to see any distinctive diffraction peak from the diffractogram of CG, confirming its amorphous nature [10], as previously seen by DSC.

The diffractogram of the CG:PEO fibers showed one main peak in the same position as the one found for the neat PEO powder. However, the decrease in relative intensity of this main crystalline peak, which was clearly visible in the diffractogram of the fibers, and the near-disappearance of the second main peak of PEO_5M_ can be described by a reduction in the crystalline phase [53] as a result of the blending of PEO_5M_ with cashew gum, which influences the organization of the crystallizable portion [44], and also for the presence of glycerol, which contributes to increasing the amorphous character of the sample [54].

Finally, the neat CG powder exhibited a low-angle diffraction peak, which in the blend appeared at an even lower angle, i.e., at around 3°. Diffraction peaks at these low angles have been previously observed and ascribed to both helical secondary structures and to other higher hierarchical assemblies [53,55]. The observation here indicates that PEO appears to influence the CG polysaccharide hierarchical assembly in a similar fashion as observed before by Vazquez et al. [31] with the FucoPol polysaccharide.

#### 3.3.3. Thermogravimetric Analysis

**Figure 6:**
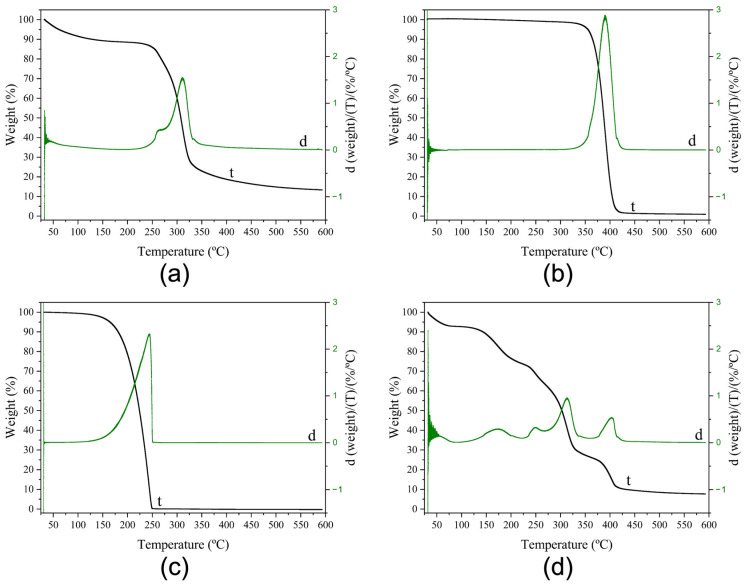
Thermogravimetric analysis (TGA) curves of (**a**) the neat CG, (**b**) the neat PEO_5M_, (**c**) the neat glycerol, and (**d**) the electrospun fibers from solution *S12*, where curve t describes the weight loss thermogram, and curve d shows the weight loss first derivative.

The thermal stability of pure materials and their blends is of paramount importance to screen potential failures during post-processes such as hot filling, humid heat sterilization, and melt compounding [56]. Thermogravimetric analysis (TGA) experiments were carried out for the electrospun fibers from solution *S12*, and for the pure CG and pure PEO_5M_. The obtained thermograms are gathered in Figure 6.

CG showed two main weight loss phases (Figure 6a). The first phase took place between 25 and 125 °C, reporting a weight loss of around 10%, which could be ascribed to sorbed water evaporation [33]. The second phase showed two maximum peaks with degradation temperature (*T*_deg_) values of 262 °C and 310 °C, reporting a weight loss of 64% due to the depolymerization of cashew gum [16]. After these two major steps, a progressive weight loss of around 10% was observed starting from 325 °C. Similar results were described by Ferreira et al. [57], who characterized cashew gum and chicha gum for applications in tablets and hydrogels production.

PEO_5M_ showed only one consistent weight loss step of 96%, showing a *T*_deg_ value of 390 °C (Figure 6b), which was also reported previously [31,58]. The neat PEO_5M_ main degradation step relates to the C–O bonds’ random chain scission [59,60].

The thermogram of the neat glycerol (Figure 6c) was also determined, in which one main weight loss step of 100% was observed, exhibiting a *T*_deg_ of 244 °C, which is in accordance with previously published results [61].

From observation of the thermogram of the electrospun fibers (Figure 6d), four clear weight loss steps were seen after an initial 10% weight loss occurred between 25 and 115 °C, which could be attributed to sorbed water evaporation as previously reported for the neat CG. The first main degradation peak was observed at a *T*_deg_ value of 173 °C and, together with the second and the third visible peaks which presented *T*_deg_ values of 249 °C and 314 °C, respectively, represent a weight loss of around 60%, attributed to the depolymerization of CG [16] and also to the degradation of the glycerol-rich phase [61]. Finally, a fourth weight loss step was observed with a maximum at 404 °C, amounting to a weight loss of around 24% as a consequence of the decomposition of PEO_5M_ and to the biopolymer thermal degradation, due to the polysaccharide side chain decomposition [62]. As previously observed [33], the addition of PEO_5M_ and glycerol contributed to reducing the overall thermal stability of *S12* fibers since the degradation temperatures took place at lower values than in the neat components.

### 3.4. ATR-FTIR Spectroscopy

The ATR-FTIR spectra of the electrospun CG-based fibers from solution *S12*, and of the pure components, that is, the neat PEO_5M_, neat CG, Span^®^ 20, and glycerol, were taken. The methodology and results can be seen in the Supplementary Materials. No apparent alterations were highlighted comparing the characteristic bands of the ATR-FTIR spectrum of the CG-based fibers to those of the neat components, suggesting the absence of detectable chemical interactions and/or degradation processes.

### 3.5. Morphology of the Fiber Mats Obtained with Multiple Emitters over a Drum Collector

**Figure 7:**
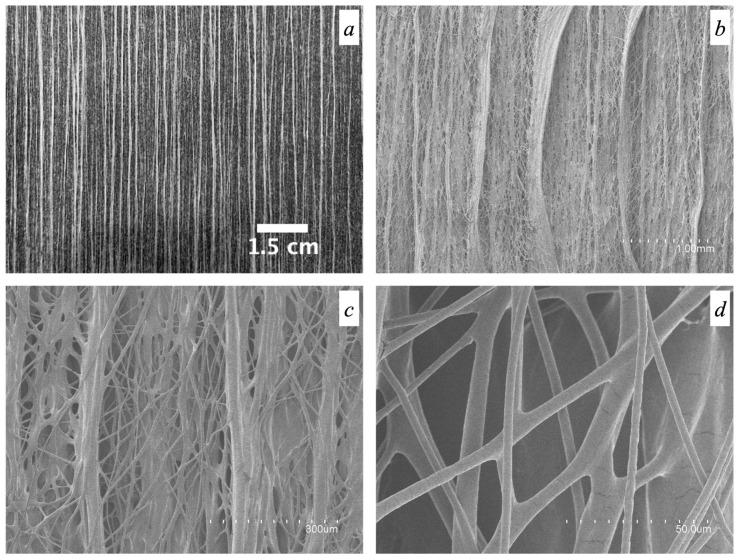
Photograph showing the naked-eye appearance of the electrospun fibers (**a**) and SEM micrographs obtained for solution *S12* using the high-throughput electrospinning process (**b**–**d**).

Figure 7 shows an optical picture of the fiber mat (Figure 7a) and the micrographs of the obtained fibers for sample *S12*, after processing using the high-throughput electrospinning setup with a five-needle injector (Figure 7b–d being micrographs of the same sample taken at different magnifications). The production yield increased from the 3.4 g/h generated in the single-emitter setup to 38.1 g/h in the high-throughput setup. As can be clearly appreciated in Figure 7a,b, the electrospun fibers generated were surprisingly aligned, even though the drum collector speed was very low with respect to yield fiber arrangement per se [63]. More interestingly, the fibers produced here bundled together while flying to the collector in the form of oriented yarns with numerous lateral thinner fiber inter-connections. To the best of our knowledge, this unexpected morphology has never been described before. This attained morphology occurred spontaneously and was not achieved by means of any fiber deposition control systems, such as additional electrostatic fields different from the one required for the jet initiation [64,65], high drum collector rotation speeds to suppress the whipping motion [63,66,67], jet-deflecting electrodes [68], or electrostatic lens as implemented by Deitzel et al. [69]. The resulting smooth fibers showed a bimodal distribution of sizes in which bundles of oriented fibers and lateral thinner interconnecting fibers are clearly visible, as already mentioned. The bundled fibers exhibited an average diameter of 45.4 ± 14.9 μm, while the lateral interconnecting fibers presented an average diameter of 4.1 ± 2.2 μm. A bimodal distribution of diameters was also reported previously by Urbanek et al. [70], who developed polycaprolactone/chitosan electrospun fibers using a drum collector rotating at 350 rpm, even though no orientation of fibers was observed.

**Figure 8:**
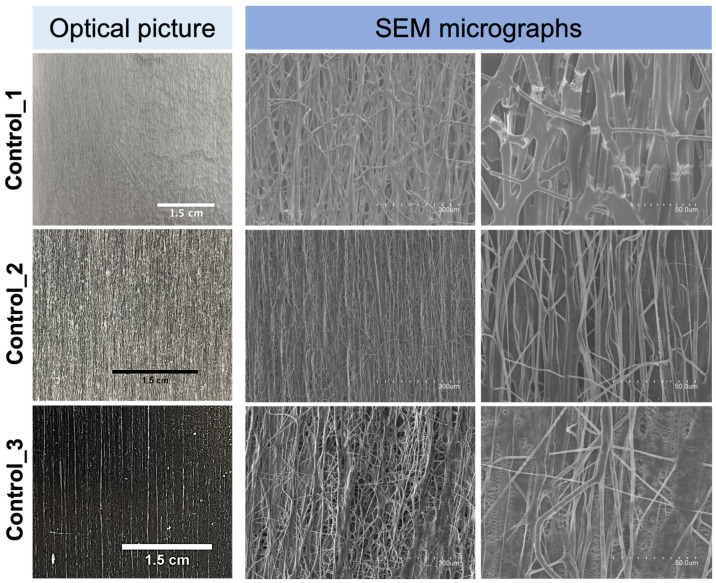
Photographs showing the naked-eye appearance and SEM micrographs of the multi-jet electrospun fiber mats from *Control_1*, *Control_2*, and *Control_3*.

With the aim of better understanding the role of both the high-molecular-weight PEO used and glycerol in the attained morphology and mechanical performance of the fiber mat from *S12*, additional experiments were carried out in the same high-throughput mode. Thus, fiber mats were produced using the polymeric solutions *Control_1*, *Control_2*, *and Control_3* (see Table 1). Figure 8 shows the naked-eye picture and the SEM micrographs of the electrospun fibers obtained from the polymeric solutions *Control_1*, *Control_2*, *and Control_3*, which can be directly compared with Figure 7. Fibers from *Control_1*, with an average diameter of 4.8 ± 2.3 μm, did show a natural material alignment trend, even though the morphology was completely different from that of the fibers in Figure 7a, i.e., thick bundles of fibers were not generated. Moreover, clearly visible cracks along the mat were seen as a consequence of the extremely high brittleness of the material during handling, which made any type of mechanical tensile testing non-feasible. As already mentioned above, in the single-emitter experiments, the presence of glycerol as in solution *S12* was considered essential to produce smooth and ductile fibers.

In the case of *Control_2* and *Control_3* fibers, a natural material orientation trend was also observed and ascribed to the high molecular weight of the PEO used, even though the morphology was not comparable to that of the blend *S12* (Figure 7). Fibers from *Control_2* and *Control_3* presented an average fiber bundle diameter of 1.3 ± 0.7 μm and 1.3 ± 0.8 μm, respectively. Furthermore, fibers from *Control_3* showed a higher degree of aggregation if compared to those of *Control_2*, which could be due to the presence of the plasticizing and hygroscopic glycerol [71] as a binding element for the flying fibers.

The above experiments (*Control_1*–*Control_3*) suggest that the particular morphology of spontaneous natural fiber alignment into very thick fiber bundles is a unique feature of sample *S12*, contributed by the high content in the mixture of the comb-like branched CG polysaccharide [72,73].

Since spontaneous aligned fibers also occur by processing high-molecular-weight PEO alone, this feature is clearly contributed by the presence of this supporting polymer. This effect on PEO has not been reported before; however, spontaneous fiber orientation has been reported before, for instance in electrospun fibers based on polystyrene of a similar high molecular weight [74]. A contribution to significant fiber bundling seems also to be promoted by glycerol (see *Control_3*), possibly as a consequence of the interactions between the CG polysaccharide and the glycerol hydroxyl groups, which bring in efficient electrostatic interactions and hydrogen bonds, leading to strong self-association [14,75] as the solvent evaporates while flying towards the collector [76]. Moreover, the presence of glucuronic acid as an end residue in the branched galactan core of CG [12,77,78,79], imparting a net polyanionic nature to the polysaccharide [12,14], may additionally contribute to the canceling out of the neat charge with the positive charges generated by the high voltage [70], thereby diminishing the overall electric field strength experienced by the biopolymer in its flights to the collector, hence helping to suppress the whipping motion and facilitating the strong bundling of the fibers. A similar canceling-out charge effect was also reported by Urbanek et al. [70] when processing chitosan/polycaprolactone fibers via electrospinning. They applied a negative polarity at the spinning nozzle that resulted in a reduction of the net positively charged chitosan and thus leading to better spinnability by reducing the likelihood of jet breaking. However, fiber orientation and bundling were not seen in the latter system.

### 3.6. Mechanical Properties

**Figure 9:**
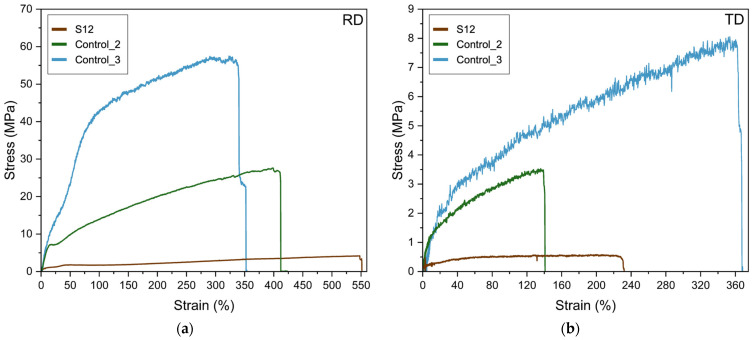
Typical tensile stress–strain curves of fibers from *S12*, *Control_2*, and *Control_3*. Both drum rolling direction (**a**) and transversal direction (**b**) are shown.

A representative example of the tensile stress–strain curves of fibers from *S12*, *Control_2*, and *Control_3* is presented in Figure 9. Table 4 displays the calculated values of the elastic modulus (E), tensile strength at break (σ_b_), elongation at break (ε_b_), and toughness (T) determined from the strain–stress curves of the high-throughput electrospun samples from *S12*, *Control_1*, *Control_2*, and *Control_3*. The attained *S12* fiber mat presented characteristics of elastomeric materials, showing in the tensile tests’ high ε_b_ and low σ_b_ values, 550% and 3.7 MPa, respectively. As can be seen, directional alignment of the bundles of fibers allowed a significantly high elongation at break, which is a unique morphological feature for *S12* fibers. The presence of the high-molecular-weight PEO is expected to contribute to achieve this superior mechanical elasticity, since *Control_2* and *Control_3* fibers have ε_b_ values of 380% and 341%, respectively. In a previous study performed by Vigani et al. [80], it was reported that the addition of small amounts of high-molecular-weight PEO (4 × 10^6^ Da), in alginate–PEO electrospun fibers, resulted in an increase from 1.5 to 10% of the mechanical elongation at break values.

Glycerol is also required to contribute to the observed mechanical properties, as can be appreciated from the impossibility to measure the mechanical properties of *Control_1* which has the same formulation of *S12* but without glycerol. The role of glycerol as a plasticizer for hydrocolloids is well documented. For instance, Martins et al. [81] reported improved mechanical performance, reaching an elongation at break value of 16%, by adding 1% *w*/*w* of glycerol when producing solution-cast CG/chitosan-based films. The role of glycerol as a plasticizing molecule for the mechanical properties of hydrophilic polymers originates through the synergetic contributions of two main phenomena, namely the reduction of the intermolecular forces between polymer chains and the subsequent promotion of chain mobility [82], and its ability to sorb moisture [83,84]. In our work, a small quantity of glycerol was able to contribute to generate a tremendously high elongation at break for the specific electrospun fiber mats developed. Indeed, higher quantities of glycerol have been usually required to achieve significant elasticity enhancements, as reported previously in the literature. For instance, Farshi et al. [85] reached an elongation at break value of 106% by adding a 12% *w*/*w* of glycerol in silk fibroin/carboxymethyl cellulose-based films. In another recent work carried out by Nagakawa et al. [86], an elongation at break value of 93% was attained by adding a 15% *w*/*w* of glycerol in poly(vinyl alcohol) nanofibrous cryogels.

The fiber mat from *S12* showed reduced mechanical properties when the analysis was performed perpendicular to the rolling direction, as seen from Table 4. This is in accordance with the morphological results seen in Figure 7, pointing to a weaker inter-fiber cohesion along the sample width.

## 4. Conclusions

To the best of our knowledge, this is the first research study tackling the production of highly stretchable CG-based materials by electrospinning. From the results, it was not possible to obtain pure CG electrospun fibers; however, electrospun fibers were successfully produced by blending CG with a high-molecular-weight polyethylene oxide supporting polymer. The resultant electrospun fibers presented a smooth and continuous structure. However, the presence of beads or spindle-like defects were also detected in the electrospun fibers. The removal of the impurities from solubilized CG by means of centrifugation, together with the use of a 3.5% *w*/*w* of pure glycerol as a plasticizer, allowed the successful generation of fibers free of both beads and spindle-like defects, as determined by scanning electron microscopy (SEM). The thermal transitions and thermal stability of the samples were assessed by differential scanning calorimetry (DSC) and thermogravimetric analysis (TGA), respectively, confirming a reduction in CG thermal stability with the addition of PEO, and a decrease in PEO crystallinity as a result of blending the polymer with CG.

In addition, when the selected optimal formula (*S12*) was electrospun over a drum collector, rotating at a very low rotational speed, from multiple emitters, very thickly aligned fiber bundles, never reported before, were obtained in the rotational direction with thinner interconnecting fibers between the bundles in the transverse direction. Such natural fiber orientation was hypothesized to be the synergic result of both the presence of small quantities of a high-molecular-weight supporting polymer, PEO_5M_, and glycerol. The obtained fiber mat presented a very impressive elastic behavior in the direction of the aligned fibers, exhibiting a tensile strength at break (σ_b_) of 3.7 MPa and an elongation at break value (ε_b_) of 550%.

Furthermore, the promising results achieved in this work together with the low cost and the high availability in nature of cashew gum, as a raw material, along with other favorable properties such as its biocompatibility and GRAS characteristics, make this biobased polysaccharide a material of interest for future research in several fields requiring non-direct aqueous contact biobased materials with elastic properties. Further research in this area holds promise for advancing elastomeric materials science and technology.

## 5. Patents

Part of the results of this study were filed in a Spanish patent application, ES16411784.

## Figures and Tables

**Table 1:** Cashew gum-based solutions prepared for the electrospinning process. TCS is the total concentration of solids solubilized in 100 mL of distilled water; CG:PEO ratio refers to the weight ratio of the two polymers included in the TCS; and CG loss after centrifuging refers to the percentage of cashew gum, in the form of impurities, removed through the centrifugation process.

Solution	TCS (% *w*/*v*)	CG:PEO Ratio	PEO *Mw* (Da)	Span^®^ 20 (% *w*/*v*)	Glycerol (% *w*/*w*)	CG Loss after Centrifuging (% *w*/*v*)
*S0*	120	100:0	-	3.00	-	-
*S1*	50	95:5	6 × 10^5^	1.00	-	-
*S2*	50	90:10	1 × 10^6^	1.00	-	-
*S3*	20	95:5	5 × 10^6^	1.00	-	-
*S4*	20	90:10	5 × 10^6^	1.00	-	-
*S5*	20	85:15	5 × 10^6^	1.00	-	-
*S6*	15	95:5	5 × 10^6^	1.00	-	-
*S7*	15	90:10	5 × 10^6^	1.00	-	-
*S8*	15	85:15	5 × 10^6^	1.00	-	-
*S9*	15	82:18	5 × 10^6^	1.00	-	<2.30
*S10*	15	82:18	5 × 10^6^	1.00	1.5	<2.30
*S11*	15	82:18	5 × 10^6^	1.00	2.5	<2.30
*S12*	15	82:18	5 × 10^6^	1.00	3.5	<2.30
*Control_1*	15	82:18	5 × 10^6^	1.00	-	<2.30
*Control_2*	2.7	0:100	5 × 10^6^	1.00	-	-
*Control_3*	2.7	0:100	5 × 10^6^	1.00	3.5	-

**Table 2:** Physicochemical properties of solutions of CG:PEO (*S8*), centrifuged CG:PEO (*S9*), and CG:PEO with 3.5% *w*/*w* of glycerol and also centrifuged (*S12*).

Sample	Viscosity (cP)	Conductivity (μS/cm)	Surface Tension (mN/m)
*S8*	24,647 ± 148 ^a^	815.1 ± 0.00 ^b^	36.0 ± 0.6 ^b^
*S9*	21,849 ± 197 ^b^	657.1 ± 0.03 ^a^	39.4 ± 1.1 ^a^
*S12*	24,189 ± 115 ^c^	645.4 ± 0.00 ^c^	37.0 ± 1.2 ^a,b^

Each column represents the mean value ± standard deviation of three independent replicas. Different letters in the same column indicate a significant difference among the samples (*p* < 0.05).

**Table 3:** Electrospinning-optimized conditions to obtain CG-based fibers with good morphology. As for voltage, the values of, respectively, the positive and negative voltage conditions used are reported.

Solution	Voltage (V+/V−) (kV)	Flowrate (μL/h)	Tip-to-Collector Distance (cm)	Needle Gauge	Fiber Formation
*S0*	+15/−0	250	12.0	27	no
*S1*	+25/−9	800	20.0	22	no
*S2*	+31/−0	500	20.0	22	no
*S3*	+29/−9	300	28.0	23	yes
*S4*	+29/−9	200	22.0	23	yes
*S5*	//	//	//	//	no
*S6*	+29/−9	400	28.0	25	no
*S7*	+29/−9	300	28.0	23	yes
*S8*	+29/−9	300	20.5	23	yes
*S9*	+22/−9	450	20.0	22	yes
*S10*	+22/−9	450	28.5	22	yes
*S11*	+22/−9	450	28.5	22	yes
*S12*	+22/−9	450	30.0	22	yes

**Table 4:** Mechanical properties in terms of tensile modulus (E), tensile strength at break (σ_b_), elongation at break (ε_b_), and toughness (T) of the electrospun fibers obtained from *S12* and *Control_1*, *Control_2*, and *Control_3* in the drum rolling direction (RD) and transversal direction (TD).

Sample	Measurement Direction	E (MPa)	σ_b_ (MPa)	ε_b_ (%)	T (mJ/m^3^)
Fibers from *S12*	RD	78 ± 23 ^a^	3.7 ± 1.4 ^c^	550 ± 54 ^a^	14 ± 5 ^c^
	TD	67 ± 39 ^a^	0.6 ± 0.1 ^c^	225 ± 17 ^b^	1 ± 0 ^c^
*Control_1*	RD	n.a.	n.a.	n.a.	n.a.
	TD	n.a.	n.a.	n.a.	n.a.
*Control_2*	RD	64 ± 3 ^a^	26 ± 2 ^b^	380 ± 46 ^b^	69 ± 12 ^b^
	TD	13 ± 1 ^a^	3.6 ± 0.2 ^b^	147 ± 18 ^b^	3.8 ± 0.6 ^b^
*Control_3*	RD	54 ± 4 ^a^	58 ± 5 ^a^	341 ± 7 ^b^	153 ± 6 ^a^
	TD	24 ± 3 ^a^	7 ± 2 ^a^	332 ± 56 ^a^	20 ± 4 ^a^

Each column represents the mean value ± standard deviation of three independent replicas. Different letters in the same column indicate a significant difference among the samples (*p* < 0.05). Statistical analysis was performed comparing samples of the same measurement direction (e.g., RD or TD).

## Data Availability

No data is available.

## Supplementary Materials

The supporting information, including Figures S1 and S2 can be downloaded at: https://www.mdpi.com/article/10.3390/polym16101355/s1 [16,81,84,87,88,89,90,91,92,93,94,95,96].
