# Supplementary material for: On the Unique Morphology and Elastic Properties of Multi-Jet Electrospun Cashew Gum-Based Fiber Mats
Source: Polymers (Basel). 2024 May 10;16(10):1355. doi: 10.3390/polym16101355 (PMC11125206; doi:10.3390/polym16101355)
Supplement: Supplementary file 1 [file polymers-16-01355-s001.zip › polymers-2940139-supplementary.pdf]

Supplementary Materials

On the Unique Morphology and Elastic Properties of Multi-jet Electrospun Cashew Gum-based Fiber Mats

Mattia Grumi[1], Cristina Prieto[1,], Roselayne F. Furtado[2], Huai N. Cheng[3,†], Atanu Biswas[4], Sara Limbo[5], Luis Cabedo[6], Jose M. Lagaron[1,*]*

[1] Novel Materials and Nanotechnology Group, Institute of Agrochemistry and Food Technology (IATA), Spanish Council for Scientific Research (CSIC), Calle Catedrático Agustín Escardino Benlloch 7, 46980 Paterna, Spain

[2] Embrapa Agroindústria Tropical, Rua Dra. Sara Mesquita 2270, Fortaleza CEP 60511-110, CE, Brazil

[3] U.S. Department of Agriculture, Agriculture Research Service, Southern Regional Research Center, 1100 Allen Toussaint Blvd., New Orleans, LA, 70124, USA

[4] U.S. Department of Agriculture, Agricultural Research Service, National Center for Agricultural Utilization Research, 1815 N. University St, Peoria, IL, 61604, USA

[5] Department of Food, Environmental and Nutritional Sciences (DeFENS), Università degli Studi di Milano, via Giovanni Celoria 2, 20133, Milan, Italy

[6] Polymers and Advanced Materials Group (PIMA), Universitat Jaume I (UJI), Castellón, Spain

* Corresponding authors: cprieto@iata.csic.es, lagaron@iata.csic.es

[†]Retired

EXPERIMENTAL SECTION

Scanning Electron Microscopy (SEM)

The morphology of the electrospun fibers was analyzed by scanning electron microscopy (SEM), using a Hitachi S-4800 microscope (Hitachi High-Technologies Corporation, Tokyo, Japan). Each sample, sized approximatively 5 mm x 5 mm, was affixed with double-side tape on the SEM sample holder. Prior to analysis, all the samples were coated with a gold/palladium alloy for 2 min using a Polaron sputter coater (Quarum Technologies, Kent, UK). During the SEM analyses, an electron beam acceleration of 10 kV was applied.

Fourier Transform Infrared Spectroscopy (ATR-FTIR) of the Samples

Attenuated Total Reflectance Fourier Transform Infrared Spectroscopy (ATR-FTIR) was performed in order to analyze potential chemical interactions between the components. ATR-FTIR spectra of the raw materials (CG, PEO, glycerol, Span® 20 surfactant) and the electrospun fibers were collected using a FTIR Tensor-37 equipment (Bruker, Rheinstetten, Germany). The samples were placed on top of the diamond crystal and appropriate contact was assured by using an ATR Sampling Golden Gate accessory (Specac Ltd., Orpington, UK). All the spectra were taken in the spectral range 600 - 4000 cm^{-1} from an average of 10 scans with a resolution of 4 cm^{-1}.

EXPERIMENTAL RESULTS

Morphology of Electrospun Fibers

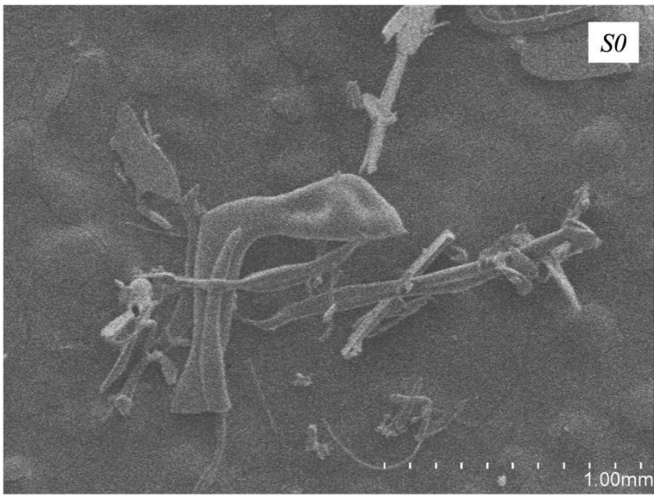

Figure S1. SEM micrograph of the electrospun material obtained from solution *S0*.

Attenuated Total Reflectance Fourier Transform Infrared Spectroscopy Analysis

The Attenuated Total Reflectance-Fourier Transform Infrared Spectroscopy (ATR-FTIR) spectra of the electrospun CG-based fibers from solution *S12*, and of the pure components, that is, the neat PEO_{5M}, neat CG, Span® 20, and glycerol, were also studied. Figure S2 shows the comparison of the ATR-FTIR spectra of these pure components and of the CG-based fibers.

CG spectrum showed a broad band at ca. 3307 cm^{-1} due to the stretching vibration of O—H groups, a small peak at 2912 cm^{-1}, attributed to the C—H stretching vibration, an absorption peak at 1635 cm^{-1}, which refers to the O—H vibrations from bound-water molecules, and a band at ca. 1620 cm^{-1}, attributed to the carboxylate group [1,2]. The peaks at 1147, 1074 and 1015 cm^{-1} were due to stretching vibrations of C—O—C from

glycosidic bonds and OH bending of alcohols [3,4]. Peaks at lower wavenumbers, such as those at 774 and 713 cm^{-1}, were ascribed to =C—H out-of-plane bending modes [5].
The PEO_{5M} spectrum showed a broad band at ca. 2862 cm^{-1}, which was assigned to CH_2 asymmetric and symmetric stretching vibrations [6]. Another broad intense peak was observed at ca. 1096 cm^{-1}, which was ascribed to the very strong C—O stretching mode of pure PEO [7,8]. Furthermore, the two adjacent peaks at ca. 950 and 841 cm^{-1} were related to CH_2 rocking and C—O stretching vibrational modes [9], respectively.
For glycerol, five characteristic bands which arise from the vibrations of C—C and C—O linkages can be seen in the spectral range from 1110- 850 cm^{-1}[10], and a broad absorption band at 3250 cm^{-1} appears that can be associated with the hydroxyl groups of glycerol [11].
Finally, Span® 20 presented characteristic bands at ca. 2922 and 2854 cm^{-1}, which can be attributed to CH_2 and CH_3 scissoring of the lipids which feature this surfactant [12].
The CG-based fibers from solution *S12* showed three characteristic bands corresponding to the peaks of the pure polymers. The first broad intense band at ca. 3300 cm^{-1} can be ascribed to the presence of sorbed water and also to the presence of polysaccharides (CG) in the sample, since it represents the O—H stretching of hydroxyls and bound water [13].
The second characteristic band can be observed at ca. 2862 cm^{-1} and it originates from the contribution of the presence of PEO_{5M} and cashew gum. This peak has been assigned to CH_2 asymmetric and symmetric stretching vibrations [6]. In the same way, the third intense band at ca. 1000 cm^{-1} is the result of the addition of the contributions of both CG and PEO_{5M} and this can explain its broader dimension if compared to the band of the two neat polymers.
No apparent changes were observed comparing the characteristic bands of the ATR-FTIR spectrum of the CG-based fibers to those of the pure components, suggesting the absence of detectable interactions and/or degradation processes.

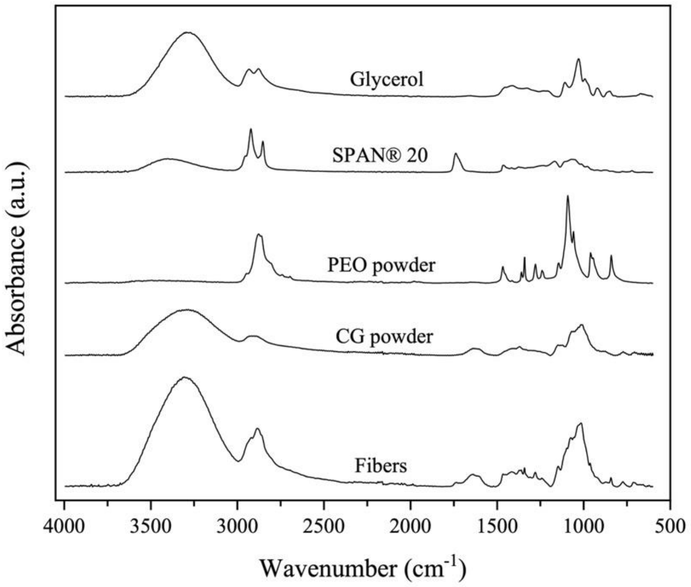

Figure S2. Typical ATR-FTIR spectra of the electrospun fibers obtained from *S12* (Fibers), pure cashew gum (CG), pure PEO_{5M} (PEO), pure Span® 20, and pure glycerol.

REFERENCES

[1] D.A. da Silva, J.P.A. Feitosa, H.C.B. Paula, R.C.M. de Paula, Synthesis and characterization of cashew gum/acrylic acid nanoparticles, Materials Science and Engineering: C. 29 (2009) 437–441. https://doi.org/https://doi.org/10.1016/j.msec.2008.08.029.

[2] F. Silva, L. Torres, L. Silva, R. Figueiredo, D. Garruti, T. Araújo, A. Duarte, D. Brito, N. Ricardo, Cashew gum and maltrodextrin particles for green tea (Camellia sinensis var Assamica) extract encapsulation, Food Chem. 261 (2018) 169–175. https://doi.org/10.1016/j.foodchem.2018.04.028.

[3] M.S. Hasnain, P. Rishishwar, S. Rishishwar, S. Ali, A.K. Nayak, Extraction and characterization of cashew tree (Anacardium occidentale) gum; use in aceclofenac dental pastes, Int J Biol Macromol. 116 (2018) 1074–1081. https://doi.org/10.1016/j.ijbiomac.2018.05.133.

[4] Y. Vázquez-González, C. Prieto, M.F. Filizoglu, J.A. Ragazzo-Sánchez, M. Calderón-Santoyo, R.F. Furtado, H.N. Cheng, A. Biswas, J.M. Lagaron, Electrosprayed cashew gum microparticles for the encapsulation of highly sensitive bioactive materials, Carbohydr Polym. 264 (2021). https://doi.org/10.1016/j.carbpol.2021.118060.

[5] C.S. Martins, D.L. Morgado, O.B.G. Assi, Cashew gum-chitosan blended films: Spectral, mechanical and surface wetting evaluations, Macromol Res. 24 (2016) 691–697. https://doi.org/10.1007/s13233-016-4103-8.

[6] A.L. Waly, A.M. Abdelghany, A.E. Tarabiah, Study the structure of selenium modified polyethylene oxide/polyvinyl alcohol (PEO/PVA) polymer blend, Journal of Materials Research and Technology. 14 (2021) 2962–2969. https://doi.org/10.1016/j.jmrt.2021.08.078.

[7] S. Ling, Z. Qi, B. Watts, Z. Shao, X. Chen, Structural determination of protein-based polymer blends with a promising tool: Combination of FTIR and STXM spectroscopic imaging, Physical Chemistry Chemical Physics. 16 (2014) 7741–7748. https://doi.org/10.1039/c4cp00556b.

[8] S. Padmaja, S. Jayakumar, Functional Group Analysis of CdS:PEO Nanocomposite Solid Films, Mater Today Proc. 5 (2018) 14473–14480. https://doi.org/https://doi.org/10.1016/j.matpr.2018.03.034.

[9] S. El-Sayed, S. Saber, A.M. el Sayed, Controlling the structural, optical, and electrical properties of PVA/PEO blend by clay nanoparticles content, Phys Scr. 96 (2021) 125812. https://doi.org/10.1088/1402-4896/ac21f5.

[10] E. Basiak, A. Lenart, F. Debeaufort, How Glycerol and Water Contents Affect the Structural and Functional Properties of Starch-Based Edible Films, Polymers (Basel). 10 (2018) 412. https://doi.org/10.3390/polym10040412.

[11] R.A. Holser, Thermal analysis of glycerol citrate/starch blends, J Appl Polym Sci. 110 (2008) 1498–1501. https://doi.org/https://doi.org/10.1002/app.27651.

[12] A.A. Khaskheli, F.N. Talpur, M.A. Ashraf, A. Cebeci, S. Jawaid, H.I. Afridi, Monitoring the Rhizopus oryzae lipase catalyzed hydrolysis of castor oil by ATR-FTIR spectroscopy, J Mol Catal B Enzym. 113 (2015) 56–61. https://doi.org/https://doi.org/10.1016/j.molcatb.2015.01.002.
[13] A. Synytsya, Fourier transform Raman and infrared spectroscopy of pectins, Carbohydr Polym. 54 (2003) 97–106. https://doi.org/10.1016/S0144-8617(03)00158-9.
